# Development and Radiological Evaluation of 3D-Printed Patient-Specific Lung Phantoms: From CT Imaging to 3D Modeling and Material Characterization

**DOI:** 10.21203/rs.3.rs-8496339/v1

**Published:** 2026-02-12

**Authors:** Ahmed M. Mortada, Jaidev Chakka, Yu Zhang, Alaa Y. Darwesh, Ayman Mokhtar Said, Sebastian John Adams, Mona H. Ibrahim, Tarek Mohsen, Mohammed Maniruzzaman

**Affiliations:** 1 Pharmaceutical Engineering and 3D Printing (PharmE3D) Lab, Department of Pharmaceutics and Drug Delivery, School of Pharmacy, The University of Mississippi, MS 38677, USA.; 2 Department of Radiology at Urology and Nephrology Center, Faculty of Medicine, Mansoura University, Egypt.; 3 Department of Physics, Faculty of Science, Zagazig University, 44516, Sharqia, Egypt.; 4 Department of Biomolecular Sciences, University of Mississippi, Oxford, MS 38677, USA.

**Keywords:** 3D printed lung phantoms, patient-specific phantoms, CT imaging, image analysis, personalized medicine, COVID-19

## Abstract

This proof-of-concept study evaluates the ability of three-dimensional printed lung phantoms to reproduce subtle pulmonary features relevant to medical imaging. An integrated workflow was developed for the fabrication and evaluation of patient-specific lung phantoms derived from high-resolution computed tomography, with emphasis on ground-glass opacities. De-identified HRCT datasets were segmented to generate both volumetric anthropomorphic lung models and standardized slim coronal phantoms, supporting reproducible imaging evaluation while reducing reliance on repeat clinical scans. The phantoms were fabricated at reduced scale using stereolithography and fused deposition modeling. Radiographic evaluation revealed clear fabrication-dependent imaging behavior. SLA phantoms exhibited smooth, isotropic microstructures and uniform grayscale response, enabling enhanced visualization of peripheral lung regions. In contrast, FDM phantoms demonstrated layered, porous architectures that produced lung-equivalent attenuation and scatter characteristics representative of pulmonary parenchyma. Quantitative grayscale analysis identified systematic differences in attenuation between fabrication methods, reflecting variations in material density and microstructural organization. Slim phantoms provided highly reproducible platforms suitable for quality assurance and quality control applications, whereas anthropomorphic phantoms preserved patient-specific anatomical detail relevant for diagnostic validation and training.

Overall, this study demonstrates that phantom geometry and fabrication strategy critically influence radiological performance and provides a flexible framework for future validation and standardized imaging research.

## Graphical Abstract

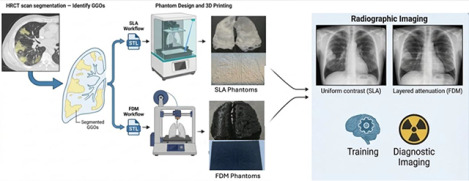

## Introduction

Anthropomorphic phantoms whether physical or computational play an essential role in modern medicine by supporting diagnostic imaging accuracy, guiding treatment planning, and enabling safe development and validation of emerging technologies.^1^ Early applications demonstrated their value in neurovascular simulation and airway management,^2^while recent systematic reviews highlight their growing modularity and adaptability across multimodal platforms.^3^ The advent of 3D printing has transformed phantom design by enabling patient specific models that replicate abnormal morphology, anatomical configuration, and spatial relationships.^3,4^ These models now extend beyond diagnostic use to drug-eluting scaffolds, cancer therapy platforms,^5^ and tissue-engineering constructs,^6^ illustrating the expanding biomedical impact of 3D-printed phantoms.

Phantoms are increasingly recognized as cross-platform solutions that integrate chemical composition with imaging performance. They have been developed for CT, MRI, ultrasound, PET, and SPECT, providing standardized surrogates for both anatomical fidelity and radiological optimization.^7^ Recent validations, such as 4D flow cardiovascular MRI using patient-specific vascular phantoms,^5^ confirm their importance for hemodynamic accuracy, while reproducibility studies in PET radiomics reveal their role in quantitative imaging stability.^8,9^ A recent 2025 study introduced programmable optical and radiological phantoms with tunable scattering and attenuation, enabling material-driven design of chemically informed imaging surrogates. Together, these examples demonstrate the versatility of phantoms across modalities while underscoring the critical role of chemical and structural composition in shaping imaging outcomes.

Among modalities, CT imaging holds particular importance for lung applications due to its ability to reconstruct volumetric representations and quantify tissue attenuation using Hounsfield Units (HU).^10^Segmentation is essential for isolating lung parenchyma and has evolved from thresholding-based methods to AI-driven approaches, though challenges remain in preserving fine features such as subpleural ground-glass opacities (GGOs).^11,12^ GGOs, especially those in peripheral and subpleural regions, are subtle radiological hallmarks of diseases including COVID-19.^13^ These lesions often lie at the limits of radiographic detectability, making them stringent test cases for phantom validation. Recent advances such as dynamic breathing phantoms^2^ and deformable anthropomorphic lung models for 4D CT^14^ illustrate how motion simulation can extend realism; however, most proof-of-concept efforts, including the present work, begin with static phantoms to establish baseline relationships between material properties and imaging performance.

Additive manufacturing methods provide distinct opportunities and challenges in phantom development. Stereolithography (SLA) enables high-resolution, isotropic structures, while fused deposition modeling (FDM) allows porosity tuning through infill design.^15–17^ Their differences in microstructural output directly impact imaging. For example, SLA resin typically yields smooth morphologies but may exhibit dimensional expansion from photopolymerization,^18–20^whereas FDM generates layered, anisotropic structures with inherent scatter heterogeneity. Despite widespread use, few studies have systematically compared how microstructure and elemental composition (e.g., Ca, Si enrichment in resins) influence radiological fidelity. Scanning electron microscopy (SEM) and energy dispersive X-ray spectroscopy (EDX) thus provide critical tools for linking material morphology and chemistry to imaging outcomes, bridging the gap between material science and biomedical imaging.^21–23^

This study addresses this gap by evaluating the chemical and structural determinants of imaging fidelity in High Resolution Computed Tomography (HRCT) derived lung phantoms fabricated via SLA and FDM, with emphasis on peripheral GGOs. Slim, film-like phantoms support dimensional reproducibility and can substitute radiographic film in quality control (QC) and quality assurance (QA) workflows for X-ray and mammography equipment,^9^ while volumetric anthropomorphic phantoms preserve anatomical fidelity for diagnostic interpretation. ISO 10993 biocompatibility remains a necessary step for applications in tissue engineering,^19^ and recent lung nodule QA phantoms illustrate how these models can be standardized for radiomics-based CT optimization.^24,25^This study builds on these trends by focusing on HRCT-derived lung phantoms, fabricated using SLA and FDM, and integrating CT, X-ray, SEM, and EDX analyses. Our objectives were to evaluate the chemical and structural determinants of imaging fidelity, with particular emphasis on peripheral GGOs as a radiologically stringent target, and to assess the potential of slim and anthropomorphic geometries for QA, diagnostic training, and future tissue-engineering applications.

## Results

### Data Collection, Segmentation and Phantom Fabrication

**Figure 1.**
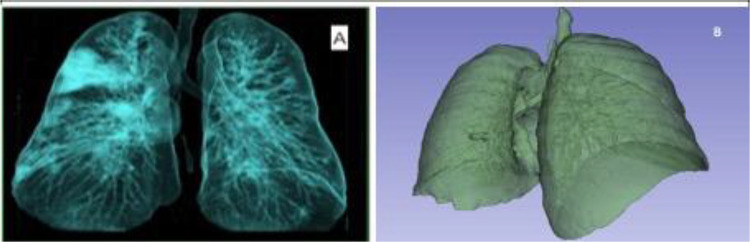
Patient-specific lung phantom geometries derived from high-resolution computed tomography (HRCT). (A) Three-dimensional segmented coronal maximum intensity projection (MIP)–based slim lung phantom designed for reproducible imaging evaluation and quality assurance applications. (B) Three-dimensional volumetric anthropomorphic lung phantom preserving patient-specific anatomy, including peripheral regions containing ground-glass opacity (GGO)–like features. These geometries represent complementary phantom designs optimized for standardization and anatomical realism, respectively.

HRCT derived lung segmentations captured both normal and diseased parenchyma, including subtle GGO (−700 to −300 HU), which served as a rigorous test for phantom fidelity.^26–28^Volumetric segmentation produced anatomically accurate anthropomorphic models, while maximum intensity projection (MIP) based approaches generated slim phantoms optimized for reproducibility (Figure 1). This dual design strategy parallels recent work where volumetric models preserved fine diagnostic structures, whereas our designed slim phantoms served as standardized surrogates for imaging validation. All segmentations were reviewed by expert radiologists prior to printing.

**Figure 2.**
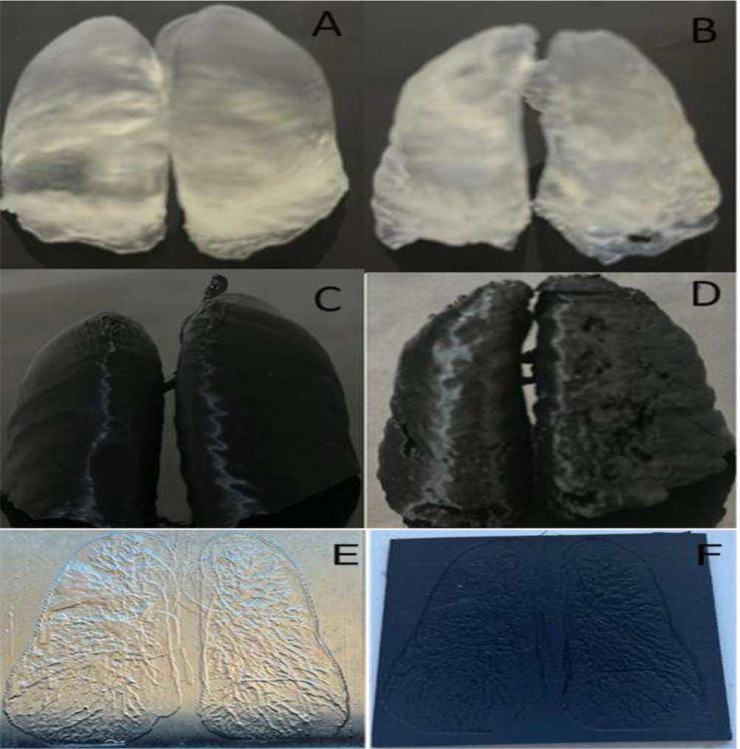
Images of fabricated lung phantoms produced using stereolithography (SLA) and fused deposition modeling (FDM). (A) Normal anthropomorphic SLA phantom. (B) Diseased anthropomorphic SLA phantom with GGO-like regions. (C) Normal anthropomorphic FDM phantom. (D) Diseased anthropomorphic FDM phantom. (E) Slim SLA phantom. (F) Slim FDM phantom.

Six lung phantoms were fabricated at a reduced 10% patient scale, representing both normal and diseased lungs (Table 1) (Figure 2). Each configuration (SLA or FDM; slim or anthropomorphic) was produced as a single sample, consistent with the scope of this study. While this limited sample size precludes robust statistical analysis, it is comparable to prior phantom feasibility studies, which have typically employed 4–8 prototypes for early-stage evaluation.^24,29–31^

Slim phantoms fabricated by SLA and FDM demonstrated measurable differences in size and weight (Figure 2). The FDM slim phantom measured 11.6 × 9.8 × 3.3 cm and weighed 25.4 g, while the SLA counterpart measured 12.02 × 9.97 × 3.35 cm and weighed 42.5 g. These results highlight two fabrication-dependent phenomena: (i) SLA phantoms exhibited ~2–3% dimensional expansion, attributable to light scattering during resin curing, a well-documented effect in stereolithography,^32–34^ and (ii) FDM phantoms showed slightly reduced edge fidelity, reflecting nozzle resolution limitations (0.8 mm) inherent to extrusion-based printing.^35^

The ~40% mass increase observed in SLA phantoms reflects their fully solid resin composition compared to the 20% infill structure of FDM prints. The higher density of PLA (1.25 g/cm^3^) relative to SLA resin (1.08 g/cm^3^) would predict heavier FDM phantoms under solid-fill conditions, but the low infill fraction yielded overall lighter constructs. These differences are not only structural but also chemical in consequence: SLA’s mass and mineral inclusions (e.g., Ca, Si) enhance X-ray attenuation, contributing to the higher grayscale contrast observed in radiographic imaging, whereas FDM’s lighter, porous structure better replicates lung-equivalent HU ranges.^36^

### Scanning Electron Microscopy (SEM) and Energy Dispersive X-ray Spectroscopy (EDX)

**Figure 3.**
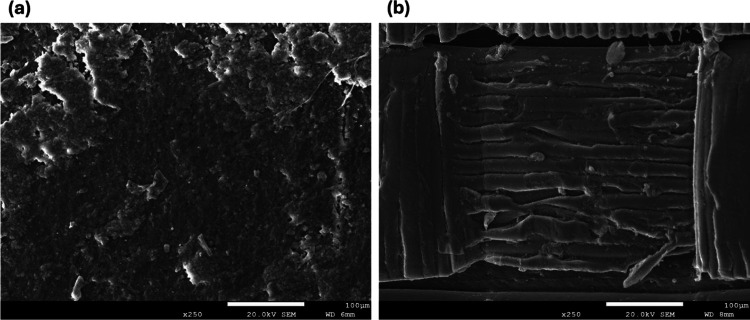
Scanning electron microscopy (SEM) images of 3D-printed lung phantoms. (A) SLA-fabricated phantom showing a relatively homogeneous surface morphology with fine porosity and isotropic microstructural features. (B) FDM-fabricated phantom exhibiting characteristic layered deposition, interlayer voids, and surface irregularities associated with extrusion-based printing.

SEM analysis performed at 250× magnification (100 μm scale bar, 20 kV; Figure 3) revealed distinct fabrication-dependent surface morphologies for the SLA and FDM phantoms. The SLA phantom (Figure 3A) displayed a relatively homogeneous surface with fine porosity and micro-voids, indicative of isotropic structural integrity. Such uniformity is advantageous for simulating alveolar microarchitecture and supports potential tissue-engineering applications. By contrast, the FDM phantom (Figure 3B) exhibited the characteristic layered deposition pattern of extrusion-based printing, including interlayer voids, cracks, and surface defects. These features correspond to an anisotropic structure with weaker interlayer adhesion, but they generate scatter heterogeneity and attenuation variability that closely mimic the complex radiodensity patterns of lung tissue.^36^

**Figure 4.**
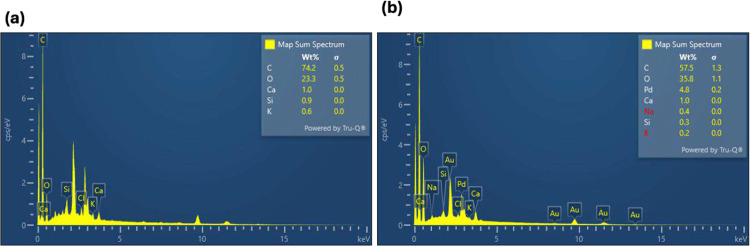
Energy-dispersive X-ray spectroscopy (EDX) spectra of 3D-printed lung phantoms. (A) SLA phantom spectrum demonstrating carbon-dominated composition with minor mineral inclusions (e.g., Ca, Si, K). (B) FDM phantom spectrum showing lower carbon content, elevated oxygen levels, and trace elemental contributions, including Au/Pd from sputter coating.

**Figure 5.**
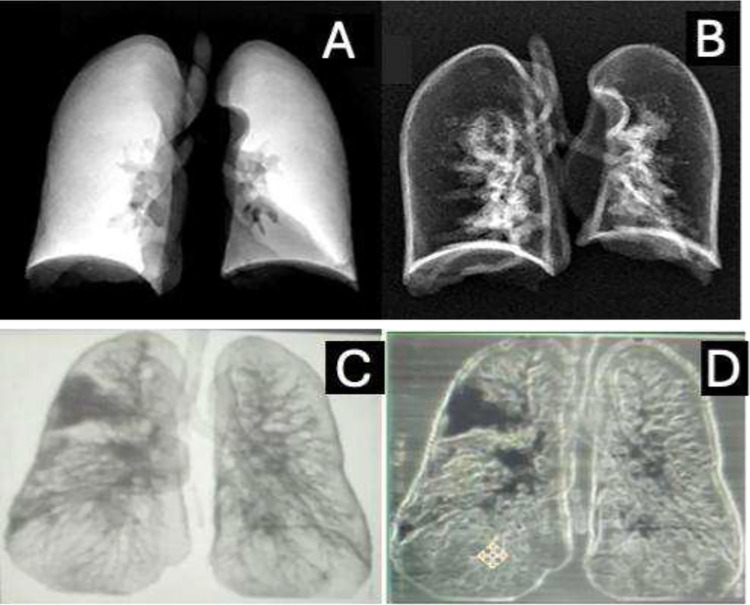
X-ray radiographic evaluation of anthropomorphic and slim lung phantoms. (A) Anthropomorphic SLA phantom. (B) Anthropomorphic FDM phantom. (C) Slim SLA phantom. (D) Slim FDM phantom.

Complementary EDX spectra (20 kV, 30 s live time, 35° take-off angle; Au/Pd masking) confirmed elemental compositions consistent with polymeric printing materials (Figure 4). The SLA phantom showed a high carbon fraction (~74 wt%) alongside minor mineral inclusions such as Ca (1.0 wt%), Si (0.9 wt%), and K (0.6 wt%), which enhance radiodensity and contribute to improved grayscale contrast in visualizing (GGOs). In contrast, the FDM phantom demonstrated a lower carbon content (~57.5 wt%) but elevated oxygen levels (~35.8 wt%), together with trace elements including Ca, Si, and Na, as well as Au/Pd peaks from the sputter coating. The increased oxygen fraction and anisotropic deposition of FDM phantoms support lung-equivalent attenuation values (–950 to −700 HU), improving their suitability for radiographic training and QA applications (Figures 4–5).^37–39^

Taken together, these SEM and EDX results demonstrate that the SLA resin’s smooth morphology and mineral enrichment favor image contrast, whereas the FDM phantom’s layered architecture and oxygen-rich chemistry optimize scatter and tissue-equivalent attenuation.

### Radiographic Evaluation and Geometry-Dependent Characteristics

Phantoms were scanned using clinically relevant exposure parameters on a digital mammography system (25–35 kV, 110–140 mAs) for slim models and a diagnostic radiography unit (40–60 kV, 50–70 mAs) for anthropomorphic models (Figure 5). These settings were adjusted to accommodate the 10% reduced patient scale, where shortened X-ray path lengths and altered scatter distribution may influence contrast relative to full-scale clinical imaging. Slim phantoms (~3.7 mm thickness) exhibited distinct material dependent radiographic characteristics. FDM models (20% infill PLA, density 1.25 g/cm^3^) necessitated higher exposures (125 mAs) than SLA counterparts (solid Elastic 50A resin, density 1.08 g/cm^3^, 100 mAs), owing to greater effective density and reduced air fraction that limited scatter heterogeneity. Conversely, SLA slim phantoms displayed superior grayscale uniformity and finer textural detail, positioning them as promising reusable surrogates for quality assurance (QA) testing in radiography and mammography systems (Figure 5C, D).

Anthropomorphic phantoms (~45 mm central thickness) further underscored fabrication-specific differences. SLA models, enriched with mineral elements (e.g., Ca, Si), exhibited elevated attenuation coefficients, requiring higher exposures but yielding enhanced conspicuity of subpleural regions critical for simulating (GGOs) seen in COVID-19 pneumonia and aligning with clinical diagnostic standards.

In contrast, FDM anthropomorphic phantoms, with 20% infill, reliably approximated lung equivalent radiodensities (~−950 to −700 HU, consistent with literature benchmarks for low-infill PLA), rendering them well-suited for dosimetry, radiotherapy planning, and diagnostic training, albeit with somewhat reduced visibility of fine subpleural features (Figure 5A,B).

Quantitative grayscale intensity analysis across apical, middle, and basal regions revealed consistently higher values in SLA phantoms (differences of +7.3% to +16.2% relative to FDM; Table 4, Figures 7–8), reflecting their isotropic structure and uniform attenuation. FDM phantoms displayed modest heterogeneity attributable to infill-induced porosity, which more closely mimicked clinical lung scatter patterns. These observations accord with the Beer–Lambert law, wherein higher density and elemental composition elevate the linear attenuation coefficient (Eq. 1). Dose compensation estimates indicated FDM required 1.20–1.30× greater exposure for equivalent grayscale output, driven by density disparities (Table 2).

Radiological review confirmed that anthropomorphic models of diseased lungs demonstrated enhanced conspicuity of peripheral and subpleural regions where ground-glass opacities (GGOs) are typically observed, a hallmark of COVID-19 pneumonia and other diffuse parenchymal processes.^40,41^ These findings are consistent with clinical radiology standards emphasizing the diagnostic importance of subtle attenuation changes in subpleural zones.

Radiological evaluation of the fabricated phantoms by experienced diagnostic consultant radiologists revealed a clear functional distinction between anthropomorphic and slim designs. Anthropomorphic phantoms, which preserve patient-specific volumetric geometry, demonstrated high diagnostic fidelity by retaining subtle morphological features such as alveolar architecture and the spatial distribution of (GGOs), making them well suited for diagnostic validation, radiologist training, and radiotherapy-related applications where accurate dose deposition depends on anatomical realism. In contrast, slim phantoms employed a standardized rectangular geometry, offering a highly reproducible platform for quality assurance (QA) and quality control (QC). Together, these findings support a complementary phantom strategy that aligns with emerging consensus favoring the combined use of anatomically faithful and standardized models for comprehensive imaging evaluation. From a practical perspective, fabrication time and material cost further differentiated the two printing techniques, with FDM slim phantoms requiring approximately 6 h at a material cost below $5, compared to approximately 8 h and $25 for SLA prints, factors that may influence scalability and clinical adoption.^42^

Although direct computed tomography (CT) scanning of the fabricated phantoms was not performed owing to their reduced 10% scale, which may alter scatter characteristics and X-ray path lengths relative to clinical conditions our radiographic findings can be contextualized against established Hounsfield Unit (HU) values reported for analogous materials in the literature. Solid prints of Formlabs Elastic 50A resin have been documented to yield approximately 39 HU, aligning closely with soft tissue or blood attenuation profiles. In contrast, polylactic acid (PLA) printed at low infill densities (e.g., 20–30%) consistently achieves lung-equivalent radiodensities in the range of −700 to −950 HU, as demonstrated across multiple studies employing infill modulation to emulate pulmonary parenchyma. The observed radiographic performance in the present work corroborates these benchmarks.

The standard Elastic 50A Resin (V04 Clear) used herein is a general-purpose photopolymer for prototyping flexible structures but lacks formal medical biocompatibility certification.

In contrast, Formlabs’ BioMed Elastic 50A Resin produced in an FDA registered, ISO 13485 facility achieves ISO 10993 compliance (cytotoxicity, irritation, sensitization) and USP Class VI certification, supporting long-term skin (>30 days) and short-term mucosal (<24 hours) contact. Given the shared formulation and mechanical properties (Shore 50A, high elongation), our observed isotropic microstructure, smooth morphology, and uniform attenuation in SLA phantoms are expected to extend to the BioMed variant, indicating potential for tissue-engineering scaffolds or patient contact models. However, device level biocompatibility (e.g., sterilization, implantation) testing was not conducted; future translational work with the certified BioMed resin is required to pursue these applications.

## Discussion

Discussion The present proof-of-concept study establishes the feasibility of patient-specific, scaled-down lung phantoms derived from HRCT data in reproducing key radiological features, particularly subtle peripheral and subpleural ground-glass opacities characteristic of COVID-19 pneumonia and other diffuse lung diseases^40,41^. Through systematic comparison of stereolithography (SLA) and fused deposition modeling (FDM), coupled with detailed physical, microstructural, elemental, and radiographic characterization, the results reveal that fabrication technique and phantom geometry profoundly govern imaging performance, yielding complementary behaviors optimized for distinct biomedical applications. Segmentation and fabrication outcomes confirmed high anatomical fidelity, with volumetric anthropomorphic models retaining patient-specific detail and slim coronal phantoms providing standardized reproducibility. Physical measurements highlighted technique-specific traits: SLA phantoms exhibited minor dimensional expansion and substantially higher mass due to solid construction and mineral inclusions, while FDM’s low-infill porosity produced lighter constructs better aligned with lung-equivalent density. SEM and EDX analyses elucidated the underlying mechanisms SLA’s isotropic, smooth morphology with Ca/Si enrichment enhanced contrast uniformity, whereas FDM’s anisotropic layering and oxygen-rich composition generated clinically relevant scatter heterogeneity and tunable radiodensity. which is complied with other researchers^37–39^ Radiographic evaluation reinforced these material-dependent differences. Although direct CT was omitted to avoid scale-induced artifacts, X-ray findings aligned closely with literature HU benchmarks (~39 HU for solid Elastic 50A; −700 to −950 HU for low-infill PLA). SLA phantoms consistently delivered superior grayscale uniformity, higher intensity (+7.3% to +16.2%), and enhanced subpleural conspicuity, albeit at higher exposures, making them ideal for precision QA and subtle feature detection. Conversely, FDM phantoms required moderately increased doses (1.2–1.3×) yet faithfully replicated pulmonary scatter and attenuation, positioning them as cost-effective options for dosimetry, radiotherapy planning, and diagnostic training. Quantitative trends conformed to the Beer–Lambert law, emphasizing the role of density and composition in attenuation. Geometry further modulated utility: anthropomorphic designs excelled in diagnostic realism and GGO spatial fidelity, as validated by expert radiologists, whereas slim phantoms offered reproducible platforms for QC workflows and potential film substitution, with practical advantages in FDM’s lower cost and time. Limited by single prototypes and reduced scale—common in early phantom feasibility—this study parallels prior work and provides a strong foundation for expansion^43–45^. Overall, radiological fidelity emerges from the interplay of microstructure, elemental composition, and design strategy. The translatability of standard Elastic 50A performance to certified BioMed variants suggests future tissue-engineering potential, pending biocompatibility testing. This framework advances additive manufacturing as a versatile tool for pulmonary imaging standardization, training, and personalized medicine, with future full-scale validation, multi-observer studies, and direct CT essential for clinical translation.

## Materials and Methods

### Ethics Approval and Consent to Participate

The study protocol was reviewed by the Urology and Nephrology Center, Faculty of Medicine, Mansoura University, Egypt. The high-resolution computed tomography (HRCT) imaging data used in this study were obtained retrospectively from the clinical imaging archives of the Urology and Nephrology Center, Faculty of Medicine, Mansoura University, Egypt. The imaging data were originally acquired as part of routine clinical care and were fully anonymized prior to their use for research purposes, with no direct or indirect patient identifiers retained. Due to the retrospective and anonymized nature of the data, informed consent was waived in accordance with institutional policies and ethical guidelines of Mansoura University. No additional imaging, intervention, or patient contact was performed for this study. All procedures were conducted in accordance with applicable ethical standards and data protection regulations.

### Materials

Two distinct materials were selected to represent structural and chemical variability in phantom fabrication. Black polylactic acid (PLA) filament (density: 1.25 g/cm^3^; tuned to 20% infill to achieve lung-equivalent radiodensity of −950 to −700 HU) was procured from MatterHackers (Lake Forest, CA, USA). This choice reflects prior reports demonstrating the suitability of PLA for radiation phantoms through controlled infill and patterning. For stereolithography (SLA), Elastic 50A Resin V04 Clear (density: 1.08 g/cm^3^; Formlabs Inc., Somerville, MA, USA) was used. The resin is reported by the manufacturer to exhibit material-level biocompatibility; however, ISO 10993-compliant device-level testing was not performed in this study. Post processing of SLA prints utilized isopropyl alcohol (99%; Fisher Scientific, UK) to remove uncured resin. The material selection was designed to contrast the porous, carbon- and oxygen-dominated polymer structure produced by FDM with the smoother, mineral-enriched resin used in SLA, which has been associated with differential X-ray attenuation.

### Clinical Dataset and Segmentation

#### Data Collection

High-resolution computed tomography (HRCT) scans of normal and diseased lungs, including cases with ground-glass opacity (GGO) patterns, were obtained from the institutional database at Mansoura University. Scanning was performed on a multi-detector CT (MDCT) system under a standardized helical acquisition protocol. Parameters included: slice thickness 1.0 mm, tube voltage 100 kVp, tube current 300 mA, and exposure time 500 ms (150 mAs effective exposure). Radiation dose indices were recorded: CTDIvol = 13.6 mGy and DLP = 435.3 mGy·cm. Scans were exported in DICOM format from the Picture Archiving and Communication System (PACS) for segmentation and modeling (Table 3). These values align with established CT imaging practices for thoracic applications.^19,20,21,22,36,41^

**Figure 6.**
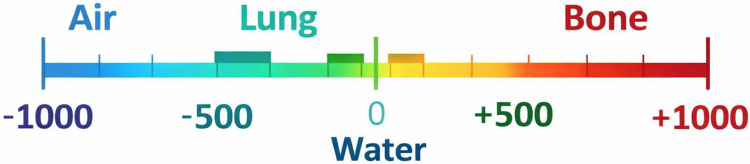
Hounsfield unit (HU) reference scale illustrating typical radiodensity ranges for major tissue classes relevant to thoracic imaging. Values shown include air (−1000 HU), lung parenchyma (−950 to −700 HU), soft tissue (−500 to −200 HU), and cortical bone (~+1000 HU). This scale provides the radiological context used to guide lung segmentation, material selection, and attenuation benchmarking throughout the study.

Segmentation was performed using AI-assisted tools followed by manual refinement in 3D Slicer and RadiAnt. A Hounsfield Unit (HU) threshold of −1024 to −250 was applied to isolated lung parenchyma and excluded mediastinal and osseous structures, consistent with established CT segmentation practice.^27,46^ HU values standardize radiodensity, with air (−1000), lung (−950 to −700), water (0), and bone (+1000).^37,47,48^ Volumetric anthropomorphic phantoms were generated directly from segmented lungs, while slim phantoms were created from coronal maximum intensity projection (MIP) images converted into STL files (Figure 6). This MIP-based approach has been applied in recent patient-specific phantom pipelines.^49^

### Phantom Fabrication (SLA, FDM)

#### Stereolithography (SLA)

SLA models were fabricated using a Formlabs Form 3B+ printer at 0.05 mm layer thickness with Elastic 50A V04 resin (1.08 g/cm^3^). Post-processing involved immersion in isopropyl alcohol (Form Wash, Formlabs, USA) to remove residual monomer, followed by post-curing for 30 min at 40 °C under UV exposure (Form Cure, Formlabs, USA). SLA printing provided isotropic microstructures and uniform contrast, though resin expansion during polymerization is a recognized source of dimensional deviation.^50,51^

#### Fused Deposition Modeling (FDM)

FDM models were prepared using Cura (Ultimaker, USA) for slicing at 0.2 mm layer height and printed on a Creality CR-30 with PLA filament (1.25 g/cm^3^, 20% infill). Printing parameters included a 0.8 mm nozzle, extrusion speed 50 mm/s, nozzle temperature 230 °C, and bed temperature 40 °C. The layered deposition yielded anisotropic structures with tunable porosity, mimicking lung equivalent.^52–54^

Fabricated anthropomorphic and slim phantoms were subjected to expert radiological evaluation to assess design-dependent imaging characteristics, while fabrication time and material consumption were recorded to enable practical comparison between printing techniques. Dimensional accuracy and mass were quantified for SLA- and FDM-produced slim phantoms using calibrated measurement tools to support fabrication-dependent comparisons.

### Scanning Electron Microscopy (SEM) and Energy-Dispersive X-ray Spectroscopy (EDX)

Given that no significant differences were expected in SEM outcomes between the anthropomorphic and slim phantoms fabricated using the same printing technique, slim phantoms were selected for SEM characterization for both methods. SEM images acquired at 250× magnification (100 μm scale bar, 20 kV; Figure 3) revealed distinct, fabrication-dependent surface morphologies between the SLA- and FDM-printed phantoms. Microstructural and chemical characterization of SLA and FDM phantoms was performed on a JEOL JSM-7200F Schottky field emission SEM equipped with an Oxford Instruments EDX detector (AZtecLive v6.1). Samples were mounted on aluminum stubs with carbon tape and sputter-coated with a thin Au/Pd (60:40) film for 60 s to enhance surface conductivity. Imaging was conducted at 20 kV, with representative morphologies acquired at 250× magnification. SEM revealed isotropic smooth morphologies in SLA and layered textures in FDM, consistent with prior phantom studies. Elemental compositions were determined semi-quantitatively from EDX spectra, highlighting carbon/oxygen predominance in PLA and calcium/silicon enrichment in SLA resin, features directly linked to X-ray attenuation.^32,33^

### X-ray Imaging

Slim phantoms (n = 2) were imaged using a mammography X-ray system (25–35 kV, 110–140 mAs), while anthropomorphic phantoms (n = 4) were imaged using a clinical radiography system (40–60 kV, 50–70 mAs). These ranges were chosen to simulate clinical acquisition conditions while preserving comparative consistency.^6,55–57^

### Lmage Analysis

X-ray attenuation was described using the Beer–Lambert law,

(Eq. 1) $$I=I_{\circ }.e^{-\mu x}$$

where *I* is the transmitted intensity, *I*_0_ is the incident intensity, *μ* is the linear attenuation coefficient, and *x* is the material thickness

While the Attenuation Coefficient can be calculated by^58^

(Eq. 2) $$\mu =\rho \cdot \mu_{\text{m}}$$

Where:

μ_m_ = Mass attenuation coefficient (cm^2^/g) (depends on the atomic number Z and photon energy)

ρ = Density (g/cm^3^)

To qualitatively account for scatter effects, an effective attenuation coefficient μ_eff_ was considered conceptually as μ reduced by scatter-related losses; this construct was used only to guide interpretation and not for quantitative calculation:

(Eq. 3) $$\mu_{\text{eff}}=\mu -\text{scatter}\;\text{loss}$$

In this study, grayscale projection intensity used as a surrogate for relative attenuation, whereas HU ranges were referenced solely as tissue-equivalence targets rather than directly measured outcomes.^37,47,59^ Profiles across superior, middle, and inferior phantom regions were analyzed for contrast uniformity.^55,60^

## Conclusion

This study demonstrates that scaled-down, patient-specific lung phantoms fabricated from high-resolution computed tomography (HRCT) data can reproduce key radiological characteristics of pulmonary parenchyma when fabrication strategy and geometry are appropriately selected. Through systematic comparison of stereolithography (SLA) and fused deposition modeling (FDM), the results show that both printing techniques yield distinct, yet complementary imaging behaviors governed by differences in microstructure, material composition, and internal architecture. SLA-fabricated phantoms exhibited isotropic microstructures, higher mass, and minor mineral enrichment, resulting in uniform grayscale response and enhanced visualization of peripheral and subpleural regions. These characteristics supported improved contrast consistency and dimensional fidelity, particularly in slim phantom geometries designed for reproducible imaging evaluation. In contrast, FDM phantoms demonstrated layered, porous architectures and oxygen-rich composition that produced lung-equivalent attenuation and scatter behavior, closely approximating reported pulmonary radiodensity ranges. Although requiring moderately higher exposure to achieve comparable grayscale output, FDM phantoms provided lightweight, cost-effective constructs suitable for applications where tissue-equivalent attenuation and scalability are prioritized. The comparison between anthropomorphic and slim geometries further highlights the importance of aligning phantom design with intended use. Anthropomorphic phantoms preserved patient-specific anatomical detail and spatial distribution of subtle features such as ground-glass opacities, whereas slim phantoms offered standardized, reproducible platforms for quality assurance and quality control tasks. Collectively, these findings establish that radiological fidelity in 3D-printed lung phantoms is jointly determined by geometry, microstructural architecture, and elemental composition. This work provides a validated framework for application-specific phantom design and supports the use of additive manufacturing as a flexible tool for imaging evaluation, training, and quality assurance in pulmonary imaging research.

## Supplementary Material

Supplementary Files

This is a list of supplementary files associated with this preprint. Click to download.

- floatimage1.png

## Figures and Tables

**Table 1.** Overview of 3D-printed lung phantoms fabricated using stereolithography (SLA) and fused deposition modeling (FDM).

	Normal case	Infected case	Total number
Anthropomorphic FDM	1	1	2
Anthropomorphic SLA	1	1	2
Slim FDM	0	1	1
Slim SLA	0	1	1

**Table 2.** Comparison of imaging-relevant properties of anthropomorphic lung phantoms fabricated by SLA and FDM.

Parameter	SLA Phantom	FDM Phantom
Density (g/cm^3^)	1.08	1.25
Internal structure	Solid	20% infill
X-ray dose (mAs)	Higher	Moderate
Attenuation coefficient	High	Moderate
Diagnostic emphasis	Subpleural GGOs	Lung equivalent HU values

**Table 3.** High-resolution computed tomography (HRCT) acquisition parameters for the clinical dataset used in lung segmentation and phantom generation.

Parameter	Value
SD (Standard Deviation)	12.50
Image Thickness	1.0 mm
Reconstruction Filter	FC03
Dose Reduction	NONE
XY Setting	2D
Samples per Pixel	1 (grayscale)
Pixel Spacing	1.628 mm
Bits Allocated	16 bits
Tube Voltage (kVp)	100
CTDIvol (Body)	13.60 mGy
DLP (Body)	435.30 mGy·cm

## Data Availability

The datasets used and/or analysed during the current study are not publicly available due to institutional and ethical restrictions but are available from the corresponding author on reasonable request, subject to applicable ethical and institutional approvals.
